# Nutrition, Periodontal Health and Birth Outcomes: An Exploratory Cross-Sectional Analysis of a Postpartum Case–Control Sample

**DOI:** 10.3390/dj14100669

**Published:** 2026-10-14

**Authors:** Valentin Bartha, Rebekka Wittlinger, Alexandra Balzer, Diana Wolff, Johan Peter Woelber, Marco M. Herz, Sara Yvonne Brucker, Sahra Steinmacher

**Affiliations:** 1 Department of Cariology, Endodontology and Periodontology, Leipzig University, Liebigstraße 12, 01403 Leipzig, Germany; 2 Department for Conservative Dentistry, Medical Faculty Heidelberg, Heidelberg University, Im Neuenheimer Feld 400, 69120 Heidelberg, Germany; 3 Department of Conservative Dentistry, Tuebingen University, Osianderstraße 2, 72076 Tuebingen, Germany; 4 Institute of Medical Biometry, Heidelberg University, Im Neuenheimer Feld 130, 69120 Heidelberg, Germany; alexandra.balzer@med.uni-heidelberg.de; 5 Faculty of Medicine Carl Gustav Carus, Policlinic of Operative Dentistry, Periodontology, and Pediatric Dentistry, TU Dresden, Fetscherstrasse 74, 01307 Dresden, Germany; johan.woelber@ukdd.de; 6 Department of Women’s Health, Tuebingen University Hospital, Calwerstr. 7, 72076 Tuebingen, Germany; sara.brucker@med.uni-tuebingen.de; 7 ART Fertility Clinics, Villa No. B22-25, Behind Marina Mall-Marina Village, Abu Dhabi, United Arab Emirates

**Keywords:** maternal nutrition, pregnancy, periodontal health, micronutrients

## Abstract

**Background and Objective:** Maternal nutrition may influence periodontal inflammation and pregnancy outcomes, yet evidence regarding food-derived nutrient intake remains limited. This study characterized maternal dietary intake relative to pregnancy-specific reference values and explored associations with periodontal and obstetric outcomes. **Materials and Methods:** In this cross-sectional analysis of a previously established case–control study, postpartum women selected according to birth outcome underwent periodontal examination and completed the German Health Interview and Examination Survey for Adults Food Frequency Questionnaire (DEGS-FFQ) 1–4 days after delivery. Periodontal examination included probing pocket depth, clinical attachment loss, bleeding on probing, and the gingival bleeding index (GBI). Exploratory associations between the nine prespecified nutrient intakes and BOP, GBI, and gestational age at delivery were assessed using Spearman rank correlations with 95% bias-corrected bootstrap confidence intervals. **Results:** The analytical sample comprised 48 women, including 15 with preterm birth; periodontal data were available for 44 participants. Reported food-derived intake was below the respective pregnancy-specific D-A-CH reference values in all participants for folate and iron, in 91.7% for iodine, 79.2% for dietary fiber, and 77.1% for niacin. Exploratory correlations between the nine prespecified nutrient intakes and BOP, GBI, or gestational age at delivery were generally small (ρ ranging from −0.092 to 0.241), and all 95% bias-corrected bootstrap confidence intervals included zero. **Conclusions:** Food-derived intake of several pregnancy-relevant nutrients was below reference values. Associations between individual nutrient intake and periodontal or obstetric outcomes were limited, with no clear evidence of associations. Given the exploratory design and small sample, these findings require cautious interpretation.

## 1. Introduction

Pregnancy is characterized by profound hormonal, metabolic, and immunological adaptations and is associated with increased requirements for several macro- and micronutrients [1]. Although international guidelines emphasize adequate nutrient intake during gestation, achieving pregnancy-specific reference values through habitual diet alone remains challenging [2,3]. Pregnancy is also accompanied by specific food-safety recommendations that may modify habitual dietary intake. In Germany, pregnant women are advised to avoid alcohol and raw animal products and to take specific precautions to reduce the risk of food-borne infections such as listeriosis and toxoplasmosis [4].

Periodontal inflammation is among the most prevalent chronic inflammatory conditions affecting women of reproductive age [5]. Gingivitis is common during pregnancy, and established periodontitis frequently persists throughout gestation [6,7]. Hormonal and vascular changes, accompanied by immune modulation during gestation, may amplify gingival inflammatory responses and thereby periodontal tissue vulnerability [8,9]. Clinically, these changes may manifest as increased gingival erythema, edema, and bleeding in response to local plaque, commonly referred to as pregnancy gingivitis. In some women, localized hyperplastic lesions such as pyogenic granuloma may also occur [10]. As a chronic inflammatory condition, periodontal disease contributes to systemic inflammatory burden and may interact with other pregnancy-related physiological processes [11,12].

Nutrition is increasingly recognized as a contextual factor influencing inflammatory status and tissue integrity [13,14,15]. Dietary patterns characterized by low intake of refined carbohydrates and high consumption of fiber-rich foods, fruits, vegetables, and micronutrients have been associated with reduced gingival inflammation [16,17], whereas diets rich in added sugars and energy-dense processed foods—also known as Western diet patterns—may promote low-grade systemic inflammation [13,18,19]. Importantly, the available literature increasingly suggests that overall dietary patterns, rather than isolated nutrients, are more consistently associated with periodontal inflammatory parameters, while evidence for individual nutrients remains heterogeneous [16,20,21,22]. In this context, a recently published cross-sectional study from a German practice-based research network demonstrated inverse associations between certain nutrients and periodontal parameters [23]. In the context of pregnancy, dietary intake-based studies indicate frequent undersupply of folate and several other nutrients, indicating that achieving recommended intakes for several pregnancy-relevant micronutrients remains challenging through habitual diet alone [24,25].

Despite growing evidence on nutrition in pregnancy and diet-related modulation of periodontal inflammation, data integrating maternal nutrient intake, dietary patterns, periodontal health, and obstetric outcomes within the same cohort remain limited. Existing studies often focus on either oral or obstetric outcomes in isolation, accompanied by heterogeneous study designs, limiting comparability across studies and complicating interpretability regarding clinically relevant associations.

Hence, the primary objective of this exploratory study was to characterize maternal food-derived nutrient intake and the extent to which pregnancy-specific reference values were reached through habitual diet alone. The secondary objective was to explore associations between individual food-derived nutrient intakes and periodontal parameters and gestational age at delivery. Given that the present analysis was derived from an outcome-selected case–control sample, the observed proportions are sample-specific and are not intended as population prevalence estimates.

## 2. Materials and Methods

### 2.1. Study Design and Setting

The present study is an exploratory cross-sectional secondary analysis of nutritional data collected within a previously reported single-center case–control study [26]. The parent study investigated periodontal status and oral microbiome characteristics in women with preterm and term birth. Participants were enrolled postpartum at the Department of Women’s Health, University of Tuebingen, Germany, between July 2018 and January 2021. Preterm birth (<37 weeks of gestation) defined the case group, whereas women with term birth constituted the control group. Thus, the sampling framework was based on birth outcome and was not intended to represent an unselected obstetric population. Eligibility was assessed after delivery, and clinical study procedures were performed within 1–4 days postpartum.

### 2.2. Ethical Approval

Reporting of this secondary analysis follows the STROBE statement for cross-sectional studies and the STROBE-nut extension for nutritional epidemiology. Completed STROBE and STROBE-nut checklists are provided as Supplementary Materials. The study adhered to the STROBE statement and received approval from the University of Tuebingen’s ethics committee (protocol number 445/2018BO). The study was registered in the German Clinical Trials Register (DRKS00026202).

### 2.3. Participants

The parent case–control study enrolled 79 postpartum women. All participants provided written informed consent before study procedures. During the study procedures, two participants, both with term birth, did not complete the full clinical examination; consequently, 77 participants contributed to the previously published clinical analysis.

For the present nutritional analysis, 48 participants had complete and evaluable DEGS-FFQ data. Thirty participants did not return the questionnaire, and one additional participant did not contribute evaluable data to the present analysis. Thus, the dietary analytical sample comprised 48 participants. For analyses involving periodontal outcomes, participants were additionally required to have the respective periodontal data available; consequently, sample size varied according to the outcome analyzed.

#### 2.3.1. Inclusion and Exclusion Criteria

Eligible participants were women aged ≥ 18 years. Exclusion criteria of the parent study included multiple pregnancies, polyhydramnios, fetal malformations, placenta praevia, severe health complications (e.g., infectious diseases, uncontrolled diabetes), and medication affecting gingival growth.

For the present nutritional analysis, availability of a complete and evaluable DEGS-FFQ was required. No participant was excluded solely on the basis of reported energy intake.

#### 2.3.2. Recruitment

**Figure 1:**
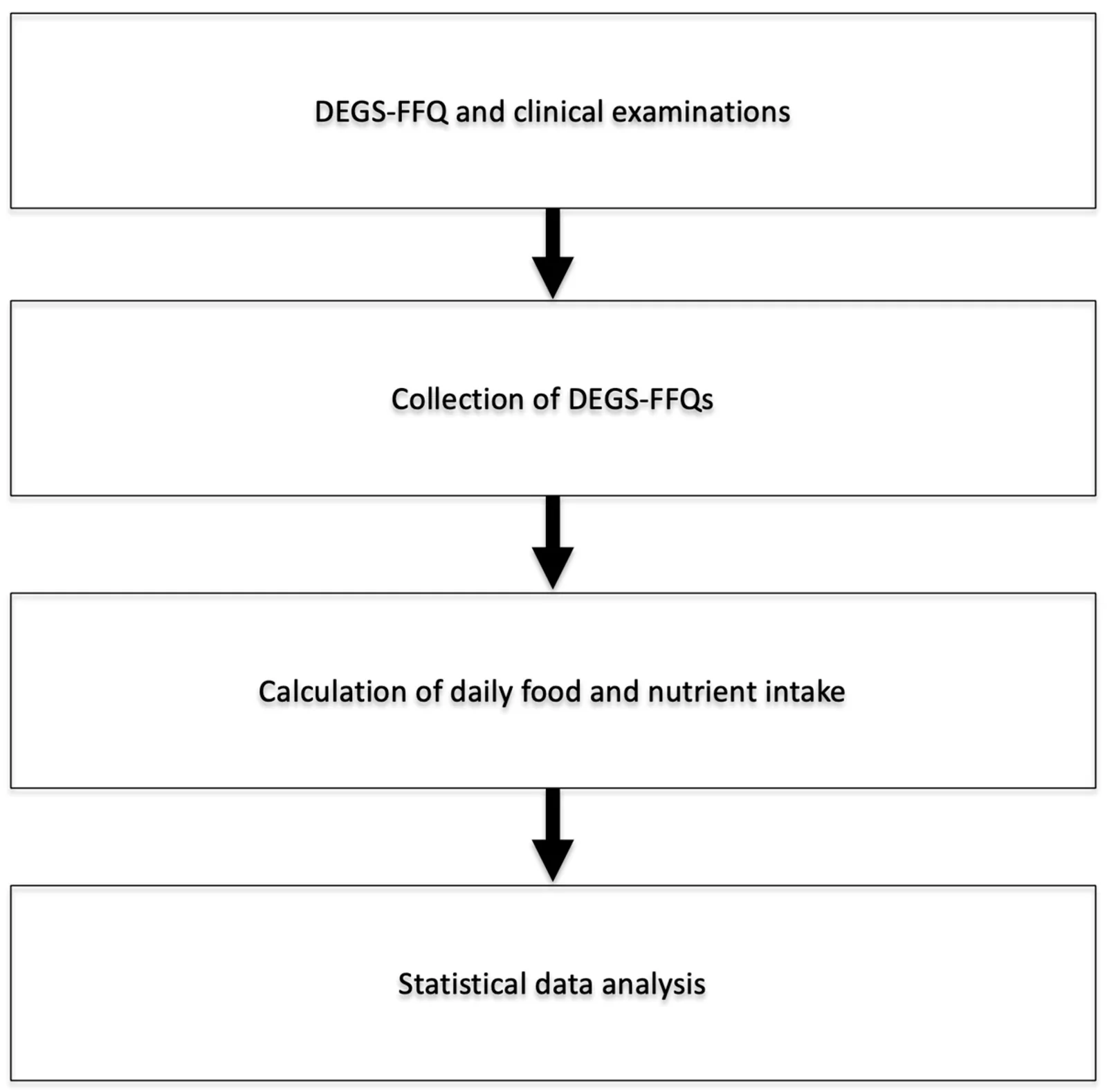
Study procedures. Note: DEGS-FFQ = German Health Interview and Examination Survey for Adults Food Frequency Questionnaire (Robert Koch Institute, Berlin, Germany).

Recruitment took place after delivery at the Department of Women’s Health, University of Tuebingen, Germany. After providing written informed consent, participants underwent the clinical study procedures during the immediate postpartum period. The German Health Interview and Examination Survey for Adults Food Frequency Questionnaire (DEGS-FFQ; Robert Koch Institute, Berlin, Germany) was distributed during the postpartum study visit and completed without interviewer assistance. The study procedures are summarized in Figure 1.

### 2.4. Data Collection and Variables

#### 2.4.1. Demographic and Clinical Data

Sociodemographic and medical data included maternal age, educational background, smoking status, medication use, gravidity, parity, mode of delivery, gestational age at delivery, and infant birth weight. General health was assessed as part of the clinical evaluation and eligibility assessment but was not systematically entered as a structured variable into the analytical dataset. Educational background was not reported by all participants.

#### 2.4.2. Oral and Periodontal Examination

Oral examinations were performed 1–4 days after delivery at the Department of Women’s Health using a headlamp and magnifying loupes. A full-mouth periodontal examination of all present teeth was performed. Probing pocket depth (PPD) and clinical attachment level (CAL) were measured at six sites per tooth using a manual periodontal probe (PCPUNC-15, Hu-Friedy, Chicago, IL, USA). PPD was defined as the distance from the gingival margin to the base of the clinically probeable sulcus or periodontal pocket, and CAL as the distance from the cemento-enamel junction to the base of the clinically probeable pocket. Probing was performed manually and gently, without additional pressure, corresponding to an intended probing force of approximately 20 g.

Bleeding on probing (BOP) was assessed at four sites per tooth and recorded dichotomously as present or absent when bleeding occurred within 10–30 s after probing [27]. Full-mouth BOP was calculated as the percentage of BOP-positive sites. Gingival inflammation was additionally assessed using the Gingival Bleeding Index (GBI).

For the present secondary analysis, periodontal diagnoses were reclassified from the original clinical data according to the 2017 World Workshop staging system, with disease stage reported independently of extent [28,29]. Consequently, both localized and generalized Stage III periodontitis were categorized as Stage III. This differs from the categorization used in the parent publication, in which generalized Stage III periodontitis was reported separately [26]. No unequivocal Stage I cases were identified in the original clinical classification. However, given the limited attachment loss defining Stage I periodontitis and its recently described clinical character as a transitional phase between gingivitis and periodontitis, the presence of localized Stage I disease among participants originally classified as having gingivitis cannot be excluded [5]. These participants are therefore reported conservatively as “gingivitis/possible localized Stage I periodontitis” in the present secondary analysis.

Two calibrated examiners performed the periodontal examinations. Examiner calibration was conducted by repeated assessment of PPD and CAL until measurements differed by no more than 1 mm. Of the 48 participants included in the nutritional analysis, four discontinued the clinical examination at different stages of the examination procedure; consequently, periodontal outcome data were available for 44 participants. Missing periodontal data were not imputed, and analyses involving periodontal outcomes were based on available cases.

#### 2.4.3. Dietary Assessment

Dietary intake was assessed using the German Health Interview and Examination Survey for Adults Food Frequency Questionnaire (DEGS-FFQ), completed without interviewer assistance 1–4 days after delivery. The DEGS-FFQ comprises 53 food groups and assesses both consumption frequency and portion size. Supplement use was not systematically assessed and was therefore not included in the nutrient intake estimates. The FFQ is a self-administered, semi-quantitative questionnaire developed for the German Health Interview and Examination Survey for Adults and has been relatively validated against two 24-h dietary recalls in German adults [30]. The questionnaire assesses the usual frequency and portion size of foods and beverages consumed during the preceding four weeks. Thus, the reference period predominantly covered late pregnancy, although it could include up to four postpartum days. Participants were not asked to reconstruct dietary intake across individual trimesters or throughout pregnancy. The DEGS-FFQ has not, to our knowledge, been specifically validated in pregnant or recently postpartum women. Reported frequencies and portion sizes were converted to average daily food-group consumption according to the procedure provided by the Robert Koch Institute. Nutrient intakes were subsequently calculated using EBISpro 2016 (Dr. Jürgen Erhardt, Willstätt-Legelshurst, Germany), based on the German Nutrient Database (Bundeslebensmittelschlüssel, BLS version 3.02), via an Excel-based calculation tool provided by the Metabolic Unit, University of Hohenheim. Nine nutrients were prespecified on the basis of their relevance to pregnancy-specific nutritional requirements and their potential relevance to periodontal inflammation and tissue homeostasis: dietary fiber, niacin, vitamin C, vitamin B12, dietary folate, iron, zinc, iodine, and calcium [1,21,23].

#### 2.4.4. Nutritional Reference Values

Reported food-derived nutrient intake was compared with the corresponding pregnancy-specific D-A-CH reference values. For each nutrient, the proportion of participants whose reported intake was below the respective reference value was determined.

### 2.5. Study Size

For this exploratory sub-study, no formal sample size calculation was performed; the study size was determined by the number of participants with complete and evaluable FFQ data (n = 48). Given the limited analytical sample, an approximate sensitivity analysis based on Fisher’s z transformation was performed to estimate the minimum detectable correlation at 80% power and a two-sided α of 0.05. The minimum detectable correlation was |ρ| = 0.412 for analyses with n = 44 and |ρ| = 0.395 for analyses with n = 48.

### 2.6. Statistical Analysis

Statistical analyses were performed at the participant level. Continuous variables were summarized as mean ± standard deviation (SD) or median with interquartile range [IQR], as appropriate, and categorical variables as n/N (%). Reported food-derived nutrient intake was summarized for the full analytical sample and descriptively stratified by birth outcome. For each of the nine prespecified nutrients, the number and proportion of participants whose reported intake was below the corresponding pregnancy-specific D-A-CH reference value were determined descriptively. No significance tests comparing nutrient intake with fixed reference values were performed.

Exploratory associations between the nine prespecified nutrient intakes and bleeding on probing (BOP), Gingival Bleeding Index (GBI), and gestational age at delivery were assessed using Spearman rank correlation coefficients. Correlation coefficients were reported with 95% bias-corrected bootstrap confidence intervals based on 10,000 resamples. Accordingly, the correlation analyses were unadjusted and are interpreted as exploratory associations.

Missing data were not imputed. Analyses were performed using complete cases, and variable-specific denominators or sample sizes are reported where applicable. As a sensitivity analysis addressing the plausibility of reported energy intake, the descriptive comparison with D-A-CH reference values was repeated after excluding participants reporting an energy intake < 4.5 MJ/day (approximately 1075 kcal/day). In addition, potential selection bias related to questionnaire nonresponse was explored descriptively by comparing participants who returned the FFQ with those who did not, using available demographic, obstetric, and periodontal characteristics.

Statistical analyses were performed using JMP, version 19 (JMP Statistical Discovery LLC, Cary, NC, USA).

## 3. Results

**Figure 2:**
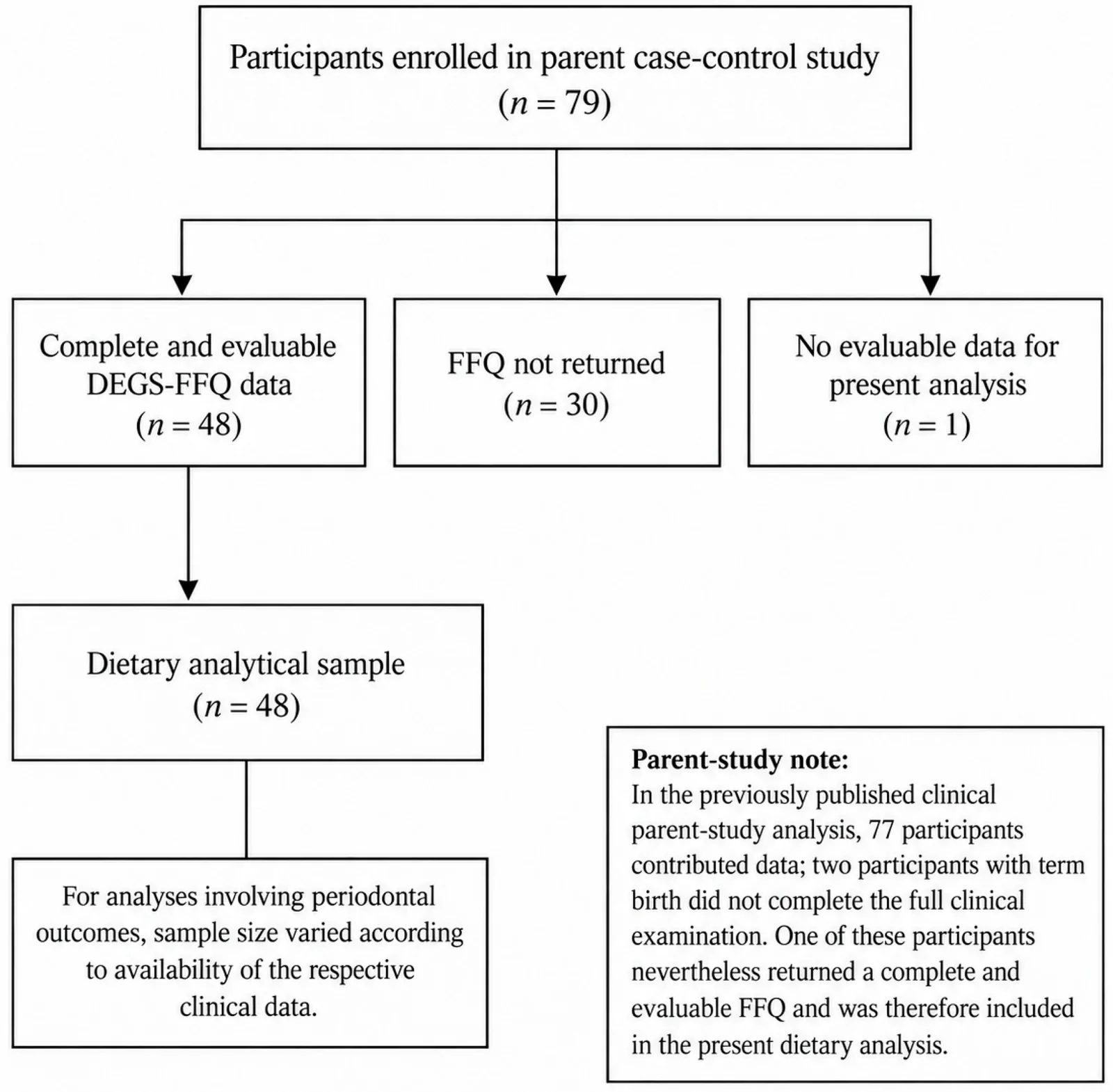
Study flow chart. Note: DEGS-FFQ = German Health Interview and Examination Survey for Adults Food Frequency Questionnaire (Robert Koch Institute, Berlin, Germany).

Of the 79 women enrolled in the parent study, 48 provided complete and evaluable FFQ data and were included in the present nutritional analysis. Thirty participants did not return the FFQ, and one additional participant did not contribute evaluable data to the present analysis. Sample sizes for analyses involving periodontal outcomes varied according to availability of the respective clinical data (Figure 2).

### 3.1. Participant Characteristics

The analytical sample comprised 48 women with complete and evaluable FFQ data, including 33 (68.8%) with term birth and 15 (31.3%) with preterm birth. Mean maternal age was 31.3 ± 5.0 years. Median gestational age at delivery was 38.93 weeks [IQR 36.21–40.36], and median birth weight was 3110 g [2720–3540] (n = 47).

Periodontal data were available for 44 participants. Of these, 5 (11.4%) were periodontally healthy, 24 (54.5%) were classified as having gingivitis, 10 (22.7%) as Stage II periodontitis, and 5 (11.4%) as Stage III periodontitis. Detailed maternal, obstetric, and periodontal characteristics, stratified by birth outcome, are presented in Table 1.

#### Nonresponse Analysis

Participants who returned the FFQ and those who did not were similar in maternal age, smoking status, and gestational diabetes. Preterm birth was more frequent among non-returners (60.0% vs. 31.3%), with correspondingly lower gestational age at delivery and birth weight. Periodontal characteristics showed a mixed pattern; non-returners had higher median CAL and a greater proportion of Stage III periodontitis, whereas BOP and GBI were lower (Supplementary Table S1).

### 3.2. Reported Nutrient Intake in Relation to D-A-CH Reference Values

Reported food-derived nutrient intake and the proportions of participants with intakes below the corresponding pregnancy-specific D-A-CH reference values are shown in Table 2. All participants reported folate and iron intakes below the respective reference values (48/48, 100%), while 44/48 (91.7%) were below the reference value for iodine. Reported intake was also below the respective reference values in 79.2% of participants for dietary fiber, 77.1% for niacin, 62.5% for zinc, and 58.3% for vitamin B12. The corresponding proportions were 50.0% for calcium and 39.6% for vitamin C.

Median nutrient intakes were generally of similar magnitude in the term- and preterm-birth groups, with no pronounced descriptive differences across most nutrients (Table 2).

#### Sensitivity Analysis

After exclusion of seven participants reporting energy intake below 4.5 MJ/day (~1075 kcal/day), 41 participants remained. As expected, median nutrient intakes were generally somewhat higher; however, the overall pattern was unchanged. The proportions of participants with food-derived intake below the respective D-A-CH reference values differed only modestly from the primary analysis, and all participants remained below the reference values for folate and iron, while 90.2% remained below the reference value for iodine (Supplementary Table S2).

### 3.3. Exploratory Associations Between Nutrient Intake and Periodontal and Obstetric Outcomes

Spearman rank correlation analyses showed no associations between the nine prespecified nutrient intakes and BOP, GBI, or gestational age at delivery (Table 3). Correlation coefficients were generally small, ranging from −0.051 to 0.241 for BOP, from −0.057 to 0.091 for GBI, and from −0.092 to 0.207 for gestational age at delivery. All 95% bias-corrected bootstrap confidence intervals included zero (Table 3).

## 4. Discussion

We found that reported food-derived intake was below the corresponding pregnancy-specific D-A-CH reference values in a substantial proportion of participants, particularly for folate, iron, iodine, dietary fiber, and niacin. Importantly, intake below a D-A-CH reference value should not be interpreted as evidence of individual nutrient deficiency or inadequate total nutrient intake; the present analysis specifically assessed the extent to which pregnancy-specific reference values were reached through habitual food intake alone. Exploratory associations between the nine prespecified nutrient intakes and BOP, GBI, or gestational age at delivery were generally small, and all 95% bias-corrected bootstrap confidence intervals included zero. Nutrient intake distributions were also broadly similar in the term- and preterm-birth groups. Taken together, our findings provide little evidence for clear associations between individual food-derived nutrient intakes and the investigated periodontal or obstetric outcomes in this exploratory sample.

Regarding obstetric outcomes, prospective cohort data have shown that food-derived intake of several pregnancy-relevant micronutrients may remain below recommended levels, while associations between individual nutrient intakes and birth outcomes have been inconsistent. However, nutrient intake distributions were broadly similar in the term- and preterm-birth groups, and we found no clear evidence of associations between any of the nine prespecified nutrient intakes and gestational age at delivery. Previous prospective studies have likewise reported inconsistent associations between individual nutrient intakes and birth outcomes [2,3,31].

We found no associations between any of the nine prespecified food-derived nutrient intakes and BOP or GBI. Correlation coefficients were generally small, In contrast, a recent German practice-based cross-sectional study using the same FFQ reported inverse associations of several nutrient intakes, including fiber, iron, zinc, and vitamin C, with periodontal parameters in patients with established moderate to severe periodontitis [23]. Differences in study populations and periodontal outcomes, however, limit direct comparison.

Pregnancy-associated gingival inflammation is influenced by hormonal, vascular, behavioral, and local clinical factors and therefore cannot be attributed to nutritional exposures alone. A recent multicenter study reported a high prevalence of gingivitis during early pregnancy [32], while periodontal pathogens have also been identified in the interdental microbiota of women during early pregnancy [33]. Direct comparison with the present cohort is limited because periodontal examination in our study was performed 1–4 days postpartum. More broadly, evidence for associations between isolated nutrients and periodontal outcomes remains inconsistent, whereas overall dietary patterns may be more informative [34].

European periodontal recommendations emphasize assessment and appropriate management of periodontal health during pregnancy; however, periodontal treatment during pregnancy, while considered safe and effective for periodontal health, has not been shown to consistently reduce adverse pregnancy outcomes [35].

We found no associations between any of the nine prespecified food-derived nutrient intakes and gestational age at delivery. Correlation coefficients were small, and all 95% bias-corrected bootstrap confidence intervals included zero. Nutrient intake distributions were also broadly similar in the term- and preterm-birth groups. Previous studies have reported heterogeneous associations between maternal nutrient exposure and birth outcomes, particularly when nutrient status was assessed using biomarkers rather than dietary intake estimates [36,37].

### 4.1. Methodological Considerations

Given that dietary intake was assessed using FFQ-derived estimates without systematic assessment of supplement use, the variables reflect food-derived intake rather than total biological exposure. Differences between estimated intake and circulating nutrient status may partly explain discrepancies with biomarker-based studies.

We found no associations between the nine prespecified nutrient intakes and the investigated periodontal or obstetric outcomes. A strength of the study is the parallel assessment of dietary, periodontal, and obstetric data within the same cohort. Nevertheless, the findings should be regarded as exploratory and hypothesis-generating rather than as evidence of clinically actionable nutritional thresholds.

### 4.2. Limitations and Strengths

Several limitations should be considered. First, the relatively small analytical sample limited statistical precision and sensitivity to detect small associations. Second, as a single-center study conducted in a tertiary care setting, generalizability to broader populations may be limited. Third, the exploratory design prioritizes hypothesis generation over causal inference. The estimates remain imprecise and should be interpreted cautiously.

Fourth, dietary intake was assessed using a food frequency questionnaire completed 1–4 days postpartum and referring to the preceding four weeks. Thus, the reference period predominantly covered late pregnancy but could include up to four postpartum days and cannot characterize dietary exposure throughout pregnancy or during specific gestational windows. The DEGS-FFQ was developed and validated in the general adult population rather than specifically in pregnant or postpartum women. Retrospective self-report in the immediate postpartum period may additionally be susceptible to recall and reporting bias.

Fifth, the present analysis was derived from an outcome-based case–control sample rather than from an unselected obstetric population. Consequently, the observed proportions of preterm birth, periodontal diagnoses, and food-derived nutrient intakes below reference values are sample-specific and should not be interpreted as population prevalence estimates.

Sixth, questionnaire nonresponse may have introduced selection bias. Of the 79 participants enrolled in the parent study, 30 did not return the FFQ and one additional participant did not contribute evaluable dietary data. Nonresponders included a higher proportion of preterm births and consequently had lower gestational age at delivery and birth weight; some periodontal characteristics also differed descriptively between responders and nonresponders. Thus, the nutritional analytical sample may not be representative of the complete parent-study population. Reasons for questionnaire nonresponse were not systematically recorded.

Seventh, supplement use was not systematically assessed. The present study therefore characterizes food-derived nutrient intake, consistent with its primary objective, but cannot determine total nutrient intake or biochemical nutrient status. Accordingly, intake below a D-A-CH reference value should not be interpreted as evidence of overall nutrient inadequacy or deficiency.

Finally, residual confounding cannot be excluded. Given the limited sample size, no multivariable confounder-adjusted models were fitted. Potentially relevant factors such as smoking, socioeconomic characteristics, oral hygiene behavior, supplement use, and other maternal or pregnancy-related factors could therefore not be comprehensively accounted for. The reported associations should consequently be considered exploratory.

However, several strengths should be noted. The study combined dietary, periodontal, and obstetric data within the same cohort, allowing these domains to be evaluated in a common clinical context. Dietary analyses were restricted to nine prespecified nutrient exposures, and periodontal outcomes were assessed using standardized full-mouth clinical examinations.

### 4.3. Implications for Research and Clinical Practice

The findings support further investigation of dietary quality during pregnancy as a multidimensional exposure rather than focusing exclusively on isolated nutrients. Future studies should employ prospective designs, systematically assess supplement use, and integrate dietary, periodontal, and obstetric data in adequately powered samples.

The present exploratory findings do not provide a basis for specific clinical nutritional recommendations or treatment thresholds. However, they illustrate the potential value of considering food-derived nutrient intake as one component of broader investigations into maternal nutrition and oral health.

## 5. Conclusions

This exploratory study showed that reported food-derived intake of several pregnancy-relevant nutrients was below the corresponding pregnancy-specific reference values in a substantial proportion of participants. Associations between individual nutrient intakes and periodontal or obstetric outcomes were generally small, with no clear evidence of associations. These findings should be interpreted as hypothesis-generating and support further investigation of overall dietary quality in adequately powered prospective studies integrating nutritional, periodontal, and obstetric data.

## Figures and Tables

**Table 1:** Maternal, obstetric, and periodontal characteristics of participants with evaluable dietary data, overall and stratified by birth outcome.

Characteristic	All (n = 48)	Term Birth (n = 33)	Preterm Birth (n = 15)
**Maternal characteristics**			
Age, years	31.3 ± 5.0	31.1 ± 4.8	31.9 ± 5.5
Current smoking, n/N (%)	5/47 (10.6)	5/32 (15.6)	0/15 (0)
**Obstetric characteristics**			
Gravidity	2 [1–2]	2 [1–2]	2 [1–3]
Parity	1.5 [1–2]	1 [1–2]	2 [1–2]
Gestational diabetes, n/N (%)	4/45 (8.9)	2/30 (6.7)	2/15 (13.3)
Gestational age at delivery, weeks	38.93 [36.21–40.36]	39.86 [38.86–40.86]	34.71 [33.43–36.14]
Birth weight, g	3110 [2720–3540] (n = 47)	3380 [3090–3665] (n = 33)	2620 [1900–2770] (n = 14)
Mode of delivery, n/N (%)			
Spontaneous delivery	19/48 (39.6)	17/33 (51.5)	2/15 (13.3)
Primary cesarean section	8/48 (16.7)	7/33 (21.2)	1/15 (6.7)
Secondary cesarean section	11/48 (22.9)	3/33 (9.1)	8/15 (53.3)
Vacuum extraction	10/48 (20.8)	6/33 (18.2)	4/15 (26.7)
**Dental characteristics**			
DMFT	6 [2–11] (n = 44)	5 [2–10.5] (n = 29)	9 [2–11] (n = 15)
**Periodontal characteristics**			
Participants with periodontal examination, n/N (%)	44/48 (91.7)	30/33 (90.9)	14/15 (93.3)
BOP, %	27.15 [15.13–44.40]	29.65 [14.68–44.65]	23.15 [15.35–43.00]
GBI, %	20.20 [8.83–35.85]	25.75 [7.78–36.68]	15.20 [13.40–35.25]
Mean PPD, mm	2.10 [2.00–2.20]	2.10 [2.00–2.20]	2.10 [1.78–2.35]
Mean CAL, mm	0.45 [0.05–1.48]	0.40 [0–0.88]	0.90 [0.30–1.93]
Sites with PPD ≥ 4 mm, %	1.19 [0–4.03]	1.12 [0–3.57]	1.49 [0.61–6.99]
Periodontal diagnosis, n/N (%)			
Periodontal health	5/44 (11.4)	5/30 (16.7)	0/14 (0)
Gingivitis	24/44 (54.5)	16/30 (53.3)	8/14 (57.1)
Stage II periodontitis	10/44 (22.7)	7/30 (23.3)	3/14 (21.4)
Stage III periodontitis	5/44 (11.4)	2/30 (6.7)	3/14 (21.4)

Note: Data are presented as mean ± SD, median [IQR], or n/N (%), as appropriate. Unless otherwise indicated, denominators correspond to the respective column total. BOP, bleeding on probing; GBI, gingival bleeding index; PPD, probing pocket depth; CAL, clinical attachment loss; DMFT, decayed, missing and filled teeth.

**Table 2:** Reported food-derived nutrient intake overall and by birth outcome, with pregnancy-specific D-A-CH reference values.

Nutrient	Pregnancy-Specific D-A-CH Reference Value	All (n = 48)	Term Birth (n = 33)	Preterm Birth (n = 15)	Below Reference Value, n/N (%)
Dietary fiber, g/day	30	18.0 [14.3–29.3]	17.0 [13.7–29.2]	19.9 [16.1–31.8]	38/48 (79.2)
Niacin, mg/day	16	11.65 [8.25–15.79]	11.46 [8.38–15.72]	12.39 [7.49–16.89]	37/48 (77.1)
Vitamin C, mg/day	105	116.9 [75.0–195.9]	119.3 [78.6–186.0]	106.5 [61.0–221.0]	19/48 (39.6)
Vitamin B12, µg/day	4.5	4.15 [2.60–5.52]	3.92 [2.69–5.35]	4.66 [1.92–5.57]	28/48 (58.3)
Folate, µg/day	550	201.9 [153.1–295.1]	198.3 [148.0–294.2]	210.7 [176.0–299.2]	48/48 (100)
Iron, mg/day	30	9.73 [7.57–13.99]	9.74 [7.36–13.37]	8.34 [7.53–14.95]	48/48 (100)
Zinc, mg/day	11 *	9.55 [7.39–13.72]	9.31 [7.40–13.69]	9.97 [6.96–15.32]	30/48 (62.5)
Iodine, µg/day	230	106.0 [82.0–149.6]	103.5 [79.6–150.2]	108.4 [83.4–148.4]	44/48 (91.7)
Calcium, mg/day	1000	1003 [751–1476]	1017 [741–1576]	989 [758–1465]	24/48 (50.0)

Note: Data are presented as median [IQR]. Pregnancy-specific D-A-CH reference values [1] are shown for comparison. Values represent recommended intakes, except for dietary fiber, which represents a guideline value, and vitamin B12, which represents an estimated value for adequate intake. The final column indicates the number and proportion of participants whose reported food-derived intake was below the respective reference value. * The zinc reference value depends on dietary phytate intake; 11 mg/day was used for the present comparison.

**Table 3:** Exploratory associations between reported food-derived nutrient intake and periodontal and obstetric outcomes.

Nutrient	BOP, ρ (95% CI)	GBI, ρ (95% CI)	Gestational Age at Delivery, ρ (95% CI)
Dietary fiber	0.075 (−0.228 to 0.360)	−0.033 (−0.351 to 0.282)	−0.082 (−0.349 to 0.205)
Niacin	0.065 (−0.222 to 0.345)	0.035 (−0.266 to 0.334)	0.018 (−0.288 to 0.330)
Folate	0.134 (−0.173 to 0.413)	0.036 (−0.304 to 0.373)	−0.010 (−0.280 to 0.273)
Vitamin B12	0.115 (−0.201 to 0.385)	0.006 (−0.325 to 0.340)	0.037 (−0.233 to 0.307)
Vitamin C	0.241 (−0.042 to 0.499)	0.071 (−0.241 to 0.386)	0.207 (−0.076 to 0.466)
Calcium	−0.051 (−0.344 to 0.228)	−0.057 (−0.357 to 0.236)	0.043 (−0.239 to 0.300)
Iron	0.110 (−0.179 to 0.384)	0.091 (−0.212 to 0.384)	−0.092 (−0.371 to 0.208)
Zinc	0.074 (−0.217 to 0.334)	0.001 (−0.309 to 0.307)	−0.046 (−0.313 to 0.235)
Iodine	0.066 (−0.234 to 0.332)	0.020 (−0.272 to 0.310)	−0.018 (−0.280 to 0.251)

Note: Values are Spearman rank correlation coefficients (ρ) with 95% bias-corrected bootstrap confidence intervals based on 10,000 resamples. Analyses were based on available cases (BOP and GBI, n = 44; gestational age at delivery, n = 48). BOP, bleeding on probing; GBI, Gingival Bleeding Index; CI, confidence interval.

## Data Availability

The raw data supporting the conclusions of this article will be made available by the authors on request.

## Supplementary Materials

The following supporting information can be downloaded at: https://www.mdpi.com/article/10.3390/dj14100669/s1, Supplementary Table S1: Characteristics of participants according to FFQ return status; Supplementary Table S2: Sensitivity analysis after exclusion of participants with low reported energy intake.

## Abbreviations

The following abbreviations are used in this manuscript:

BOP	Bleeding on probing
CAL	Clinical attachment level
CI	Confidence interval
D-A-CH	Germany–Austria–Switzerland reference values
DEGS-FFQ	German Health Interview and Examination Survey for Adults Food Frequency Questionnaire
DGE	German Nutrition Society (Deutsche Gesellschaft für Ernährung)
DMFT	Decayed, missing, and filled teeth
DRKS	German Clinical Trials Register
FFQ	Food frequency questionnaire
GBI	Gingival Bleeding Index
IQR	Interquartile range
PPD	Probing pocket depth
PTB	Preterm birth
SD	Standard deviation
STROBE	Strengthening the Reporting of Observational Studies in Epidemiology
