# Supplementary material for: Nutrition, Periodontal Health and Birth Outcomes: An Exploratory Cross-Sectional Analysis of a Postpartum Case–Control Sample
Source: Dent J (Basel). 2026 Oct 14;14(10):669. doi: 10.3390/dj14100669 (PMC13703504; doi:10.3390/dj14100669)
Supplement: Supplementary file 1 [file dentistry-14-00669-s001.zip › dentistry-4534977-supplementary.pdf]

Supplementary Tables

Supplementary Table S1. Characteristics of participants according to FFQ return status.

Characteristic	FFQ returned (n = 48)	FFQ not returned (n = 30)
Age, years	31.3 ± 5.0	31.4 ± 5.7
Current smoking, n/N (%)	5/47 (10.6)	3/26 (11.5)
Gravidity	2 [1–2]	1 [1–2]
Parity	1.5 [1–2]	1 [1–2]
Preterm birth, n (%)	15/48 (31.3)	18/30 (60.0)
Gestational age at delivery, weeks	38.93 [36.21–40.36]	36.36 [35.61–38.90]
Birth weight, g	3110 [2720–3540] (n=47)	2785 [2443–3388]
Gestational diabetes, n/N (%)	4/45 (8.9)	2/27 (7.4)
BOP, %	27.15 [15.13–44.40] (n=44)	17.60 [12.28–26.25] (n=26)
GBI, %	20.20 [8.83–35.85] (n=44)	12.50 [7.25–19.09] (n=28)
Mean PPD, mm	2.10 [2.00–2.20] (n=44)	2.15 [2.10–2.38] (n=26)
Mean CAL, mm	0.45 [0.05–1.48] (n=44)	1.40 [0.38–1.95] (n=26)
Sites with PPD ≥4 mm, %	1.19 [0–4.03] (n=44)	3.03 [1.19–6.55] (n=26)
**Periodontal diagnosis, n/N (%)**		
Periodontal health	5/44 (11.4)	2/26 (7.7)
Gingivitis	24/44 (54.5)	10/26 (38.5)
Stage II periodontitis	10/44 (22.7)	7/26 (26.9)
Stage III periodontitis	5/44 (11.4)	7/26 (26.9)

Supplementary Table S2. Sensitivity analysis after exclusion of participants with low reported energy intake.

Nutrient	Primary analysis, below reference value n/N (%)	Sensitivity analysis*, below reference value n/N (%)
Dietary fiber	38/48 (79.2)	31/41 (75.6)
Niacin	37/48 (77.1)	30/41 (73.2)
Vitamin C	19/48 (39.6)	14/41 (34.1)
Vitamin B12	28/48 (58.3)	22/41 (53.7)
Folate	48/48 (100)	41/41 (100)
Iron	48/48 (100)	41/41 (100)
Zinc	30/48 (62.5)	23/41 (56.1)
Iodine	44/48 (91.7)	37/41 (90.2)
Calcium	24/48 (50.0)	18/41 (43.9)

Note: Sensitivity analysis excluding seven participants with reported energy intake <4.5 MJ/day (approximately 1075 kcal/day). Values indicate the number and proportion of participants whose reported food-derived nutrient intake was below the corresponding pregnancy-specific D-A-CH reference value. Reference values and their classification are provided in Table 2 of the main manuscript.
